# View From Outside the Viewing Sphere

**DOI:** 10.1177/2041669518774806

**Published:** 2018-05-23

**Authors:** Jan Koenderink, Andrea van Doorn, Robert Pepperell

**Affiliations:** Justus Liebig Universität Giessen, Germany; University of Leuven (KU Leuven), Belgium; Utrecht University, The Netherlands; Justus Liebig Universität Giessen, Germany; Utrecht University, The Netherlands; Cardiff Metropolitan University, Wales, UK

**Keywords:** panoramic vision, visual space, pictorial space

## Abstract

The ‘viewing sphere’, as defined by Euclid and explored by Gibson as the ‘optic array’, is generally thought of as wrapped around the eye. Can an observer step out of it? With currently popular photographic techniques, the spectator is forced to, because the viewing sphere is presented as a pictorial object. Then the question is whether human observers are able to use such pictorial representations in an intuitive manner. Can the spectator ‘mentally step into the interior’ of the pictorial viewing sphere? We explore this issue in a short experiment. Perhaps unsurprisingly, because the eye cannot see itself, the short answer is no.

## Introduction

Consider Euclidian space $E^{3}$. For simplicity, we take the origin O at the eye. Then any point $P\in E^{3}/\{O\}$ (Euclidian space with the origin deleted) defines a ‘visual ray’ **P**, the directed line segment OP. This is Euclid’s (around 300bce [Burton, 1945]) model of optical space. Euclid augments it with the notion of a limited density of rays, or – equivalently – a finite ‘thickness’ of the rays, say about a square minute of arc solid angle for the human observer.

Euclid’s theory is a theory of *optical information*. It is quite powerful in that ray counting lets one derive the modern expressions for the resolving power of microscopes or telescopes and so forth (Koenderink, 1982). Generically, Euclid’s Optics is either taken for a flawed treatise on physical optics (Gross, 1999) or as an equally flawed treatise on linear perspective (Pirenne, 1971), either view reflects a misinterpretation.

It is common to introduce a map $\Pi :E^{3}/\{O\}\to S^{2}$ of the optical space to the unit sphere, simply setting $p\in S^{2}=P/\|P\|$, where ‖P‖ denotes the length of the segment OP. This yields the ‘viewing sphere’ $S^{2}$. The viewing sphere is a formal, ideal object (Van Brummelen, 2013) that has no special significance in physical optics. One often uses it as a parameter space, specifying points of $S^{2}$ as labels for visual directions.

The sphere is conventionally parameterized by means of a pair of angular coordinates {ϑ,ϕ}. Here, ϑ is the ‘elevation’, or height above the horizon. It takes values in the range $\{-90^{\circ },+90^{\circ }\}$, with $\vartheta =+90^{\circ }$ specifying the zenith, $\vartheta =-90^{\circ }$ specifying the nadir. The angle ϕ denotes the ‘azimuth’, that is, the angle reckoned from the forward direction along some small circle centred on the zenith or nadir to the current view direction. Thus one defines ϕ=ϑ=0 for the principal direction of view and $\phi =180^{\circ },\vartheta =0$ the direction towards the rear, the antipode of the principal fixation point, called ‘occipital pole’ by Helmholtz.

The viewing sphere is quite large, its spherical area measures about 210,000 times the area of the full moon. It contains about 200 million visual rays in the sense of Euclid.

Gibson (1950, 1966, 1979) wrestled with the ontological status of the ‘optic array’ for many years, but never wholeheartedly accepted its status as merely formal, ideal object. At first, he spoke of a ‘one-inch sphere’, apparently identifying the viewing sphere with the eye ball. (Later, Gibson switched to the more abstract notion of ‘nested solid angles’. However, this relates to the point-topology of the optic array and has no relation to the present issues.)

Recently a variety of cameras that allow one to capture the full optic array in a single exposure has become available to the general public. These are optical machines that deliver a pictorial representation of the viewing sphere. Such photographs have an obvious appeal, people look at planispheric and rolling globe pictures on their smartphones. So-called small earth circular images are frequently seen in the popular media, so are cylindrical projections that may be scrolled by the user (Koenderink & van Doorn, 2017). The popularity of such renderings is partly due to the fact that they are unusual and thus (at least for the time being) have a gimmicky character. The fact that various representations are used perhaps suggests that none is an obvious ‘winner’, there is no way such optical data are intuitively and uniquely ‘represented right’.

Does this novel technology extend the power of the eye or is it a mere gimmick? It would be a potential ‘eye opener’ if people could read the pictures intuitively, without falling into major misperceptions. It is not a priori evident that this would be unproblematic. For instance, observers estimate the extent of their visual fields (Koenderink, van Doorn, & Todd, 2009) as being much smaller than it is (common), or much larger than it is (less common, but not infrequent). For most observers, the space behind their backs does not exist (Attneave & Farrar, 1977; Phillips & Voshell, 2009). One takes an optic hemisphere for all there (optically) is. Thus, there is some reason to expect problems due to topological factors.

Conventional pictures almost without exception depict (much) less than the halfspace in front of the observer. Photographs and drawings depicting more than about 90° usually lead to complains that they are ‘distorted’ (despite the perfect perspective) and are usually avoided by professional photographers, whereas artists intentionally use formally incorrect perspective in order to make their depictions ‘look right’. Attempts to show more than a hemisphere of the optic array are rare, exceptions being panoramas, where the viewer is inside the physical picture. In modern Western art, Picasso did experiments in the 1950s and Flocon showed a planar representation of a full optic array (final figure of the book; Flocon & Barre, 1968). Such experiments were only appreciated by a very minor audience though.

In a recent experiment (Koenderink & van Doorn, 2017), using the common planispheric rendering, we found that essentially all observers commit characteristic topological errors. They do not recognize the fact that the horizon is a topological circle, ignoring both the connectivity and the special status of antipodal directions (Koenderink & van Doorn, 2017; Koenderink, van Doorn, & Lappin, 2003). Since all observers also commit huge errors of a metrical nature, this leads to major misperceptions of the depicted scene.

In this study, we focus on the so-called *rolling ball representation*. This is a popular representation of the optic array ‘as seen from the outside’, usually presented in rotation. In some implementations, the user may control the rotation on a touch screen of a smartphone say.

**Figure 1.**
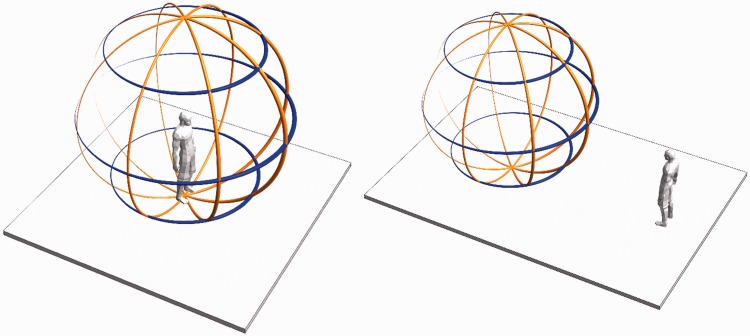
This explains the cases of ‘viewing from the inside’ and ‘viewing from the outside’. At left, the case of viewing from the inside, as when you experience the optic array. At right, the case of viewing from the outside, as when you look to an earth globe. Although you cannot see your optic array from the outside, you can view a spherical rendering of it from the outside. This happens when viewing a celestial sphere, for instance. We discuss these cases in detail in the discussion section.

Since one commonly enjoys the optic array from the *inside*, problems may be anticipated when it is presented as a picture so as to be seen from the *outside*. The question is *are spectators able to mentally step into the inside of the pictorial viewing sphere*? (See Figure 1.) That is to say, are they able to put their mental eye at the position of the camera? That is not at all trivial because all pictures are infinitely ambiguous. For instance, in the rolling ball presentation, one possible interpretation is an actual rolling ball, painted at its outside surface, like a photograph can be seen as a planar surface covered with pigments. In the latter case, many people would hold that you cannot only look *at* a photograph but also look *into* a photograph. One meets a similar problem in the case of the rolling ball interpretation. It is well known that the ability to look *into* a picture is typically human and apparently learned. Here, we report empirical data on this issue.

## Methods

### Stimuli

We used a Ricoh Theta S camera (Ricoh, 2016) to take the photographs. This camera has a pair of back-to-back fisheye lenses, each covering a field of 190°. Because the two cameras are rigidly connected, stitching is unproblematic, it is handled in-camera. The camera can be remote controlled by way of an iPhone app. A single exposure yields a 5,376 × 2,688–pixels image, covering the full optic array with a resolution of about 4' at the horizon. We use such photographs as texture maps on a computer graphics triangulated sphere.

Photographs were taken on an overcast day. This was done to avoid shading and cast shadows due to the (unidirectional) sunbeam, as these would introduce a fixed absolute direction.

**Figure 2.**
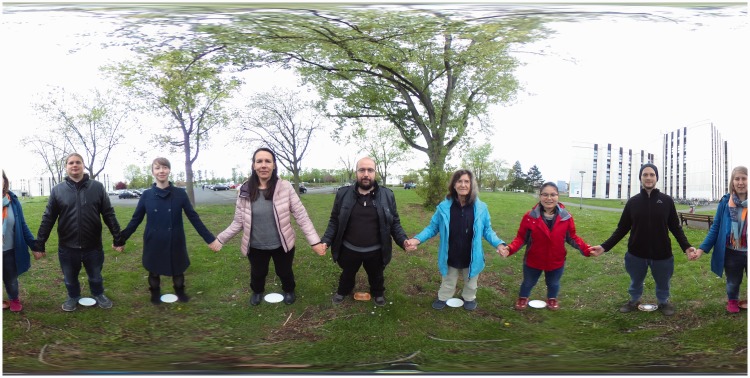
This is a planispheric image of the actors looking inwards. In this configuration, the actors – arranged in a circle – face each other. Notice how the outmost person is ‘split’ and appears both at the left and the right edge.

**Figure 3.**
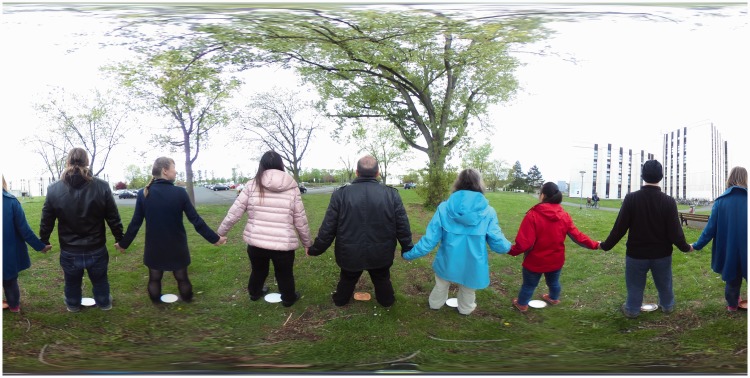
This is a planispheric image of the actors looking outwards. In this configuration, the actors – arranged in a circle – turn their backs to each other.

We set up two scenes. In each scene, eight actors were uniformly distributed over a circle centred on the camera. The radius of the circle was 1 m. The scenes are illustrated in Figures 2 and 3. Both pictures represent the so-called equirectangular maps (Fr.: plate carrée, G.: quadratische Plattkarte), introduced by Marinos of Tyros (ca. 100ce; Snyder, 1993). The vertical dimension is the elevation ϑ, the horizontal dimension is the azimuth ϕ. Thus, the full upper edge of the picture is a (very singular) map of the zenith, the full bottom edge of the picture is a (very singular) map of the nadir. The left edge and the right edge formally coincide. Both edges contain the occipital pole.

In one scene, all observers faced the camera (with body, head and eyes), in the other, they had their backs to the camera. In both cases, the actors joined hands, so they are obviously mutually connected. Note that when the actors face each other, they also face the camera, whereas when turning their backs to each other, they look away from the camera.

In the final presentation, the photographs were presented as texture on a rotating sphere on a computer screen (see supplementary material). The precise layout is discussed later in this article, since it is closely related to the design of the experiment. The display was the screen of an Apple MacBook Pro 15–inch (mid-2012), the sphere subtended a diameter of 8 cm on the screen. Viewing was in an informal setting at comfortable viewing distance (ca. 50 cm). The display thus measured about 35° of visual angle wide and 23° high. We have no reason to believe that these measures are essential.

### Participants

Participants were as follows:

- PhD students, postdocs and staff at the laboratory of experimental psychology at the Justus-Liebig Universität, Giessen, Germany, tested by Andrea van Doorn. Most observers were in their 20 s to 30 s. Total 21:10 males and 11 females.
- PhD students, postdocs and staff at the laboratory of experimental psychology at the University of Leuven (KU Leuven), Leuven, Belgium. Most observers were in their 20 s to 30 s. Total 31:17 males and 14 females.
- Observers of various ages tested during a workshop in Spain by Robert Pepperell. Variety of ages. Total 22:15 males and 7 females.
- Students of various ages from the Cardiff School of Arts tested by Robert Pepperell. Variety of ages. Total 11:4 males and 7 females.

In total, there were 85 participants.

Distribution over genders was about equal (45% female). The 21 Giessen participants were aware that we were experimenting with a full optic array camera, the others did not. It made no difference to their result though.

## Experiment

This experiment is hardly regular psychophysics, but might perhaps be classified ‘experimental phenomenology’, since we essentially collect first person reports.

Although one objective was to perform the experiment in an informal setting, typical for generic use, it is clearly desirable to exert some control. Thus, the design of the task is of crucial importance. The next section is devoted to that.

### Design of the Task

A session can be divided into various mutually independent stages, visited in sequential order. At each stage, one needs to control the viewing of the participant and perhaps to instruct the participant on the next stage. Stages that we formally distinguished are:

Registration: This is a purely administrative stage in which some identifying data of the participant are collected. It is not further discussed;
Instruction: This is a very important stage in which the participant is made aware of what to expect and what to look for;
Presentation: This is the actual stimulus presentation. Here, it is needed to force the participant to look in the intended manner;
Responding: At this stage, the participant is asked for a response. It is a critical stage in that it is hard not to impose constraints that might influence the result;
Questioning: At this stage, the actual session is over. However, one might ask the participant various questions (in this experiment we use only one). The generic techniques of questionnaires apply.

We proceed to describe the essential choices that were made in these cases. It is evident that such choices might make a difference. We will hardly attempt to generalize beyond them.

#### Registration

Because the task involves a non-trivial spatial judgement, we reckoned it perhaps relevant to consider gender a possible parameter, at least requiring a check. Names were registered because they might allow reference to the previous experiment (Koenderink & van Doorn, 2017) in case some singular result occurred. However, the groups of participants only partly coincided. Possible overlap is limited to the Giessen group anyway.

In retrospect, neither age nor gender made a difference. In order to show these up, a much larger sample from the generic population would have to be taken into account.

#### Instruction

**Figure 4.**
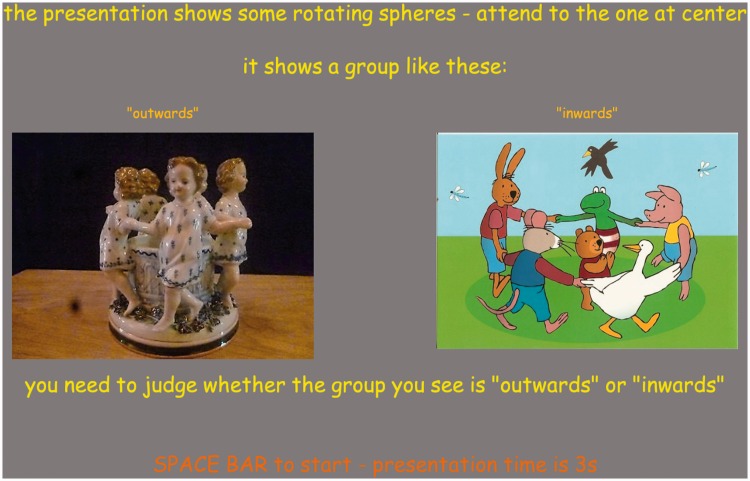
The instruction screen. At left a group looking outwards, at right a group looking inwards.

The instruction was implemented as a full screen page (Figure 4). It contained some text and two images, placed so as to be read as three separate messages.

The top line reads [the presentation shows some rotating spheres – attend to the one at center] and right below [it shows a group like these:].

Right below these lines, we placed two images, one marked [“inwards”], the other [“outwards”]. These images showed three-dimensional configurations of figures placed in a circle, either facing inwards or outwards. In either case, the viewpoint was conventional, that is, from outside the group, a slightly raised position. These images (and their identifying words) were placed in random left/right order. It was expected that the images would render the terms ‘inside’ and ‘outside’ clear. Reviewers of the manuscript judged that this made the outcome of the study entirely trivial. We obviously disagree and leave it to the reader to decide. We could have changed the response mode (see below) to a drawing of a ground-plan of the physical configuration, including the camera. We decided against this because (as we know from experience) this takes much effort, many participants complaining that they ‘cannot draw’. The present choice appeared natural enough to all participants, we do not feel that there was any confusion or that we intentionally biased the participants to some particular choice.

Below the images was the instruction [you need to judge whether the group you see is “outwards” or “inwards”]. Below that (in less emphasized type due to colour) was [SPACE BAR to start - presentation time is 3 s].

*The rationale* was as follows. We wanted to prepare the participants to the fact that they would be presented with spherical pictorial objects and that there were several of these and that they should focus on the central one – *without showing them an example*. We also wanted to prepare them to the fact that they would have a fairly short presentation, 3 seconds is only a ‘good look’ worth. The remark about the space bar alerted them to the fact that they could self-initiate the presentation. This is very important indeed, because a good look is short, but long enough when anticipated.

The images defined the task: *Are the figures in the group I’m going to see facing inwards or outwards?* The randomization of position was a precaution against systematic errors. The random choice was written to the response file for later checks, although it was not anticipated to be relevant.

#### Presentation

**Figure 5.**
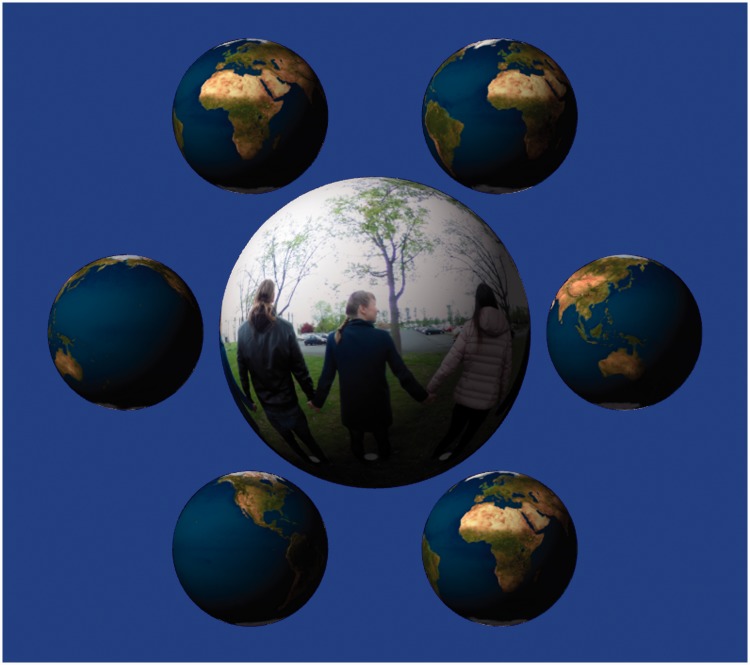
This is a screen shot in the presentation mode.

This is the actual stimulus presentation (Figure 5). The full screen had the stimulus at centre, with a group of six auxiliary objects around it. The stimulus was the ‘rolling ball’ presentation of the picture of a scene, the auxiliary objects were small presentations of a rotating earth globe. The globes were half the size of the rolling ball picture. All spheres (seven in all) rotated simultaneously about the vertical at the same speed and in the same sense. In the 3 seconds of presentation, the spheres completed about a third of a full turn. The phases of all spheres were random in any presentation.

All spheres were shown as lightly shaded, as if illuminated from a direction to the high left with respect to the observer.

*The rationale* was as follows. First of all, since we were interested in momentary visual awareness, not perception in the course of reflective thought, we limited the presentation to a few seconds. Three seconds is amply sufficient for a good look, at least if the presentation is self-initiated, but it is too short for scrutiny.

Second, when viewing a rolling ball presentation, experienced observers can – given time – invert the sphere and look into a concave hemispherical cup instead of a convex full sphere. This would defeat our purpose, so this viewing mode needs to be counteracted. We used a number of devices to do that. The shading certainly helps to make the spheres appear solid and convex. The earth globes are not easily inverted and a group of spheres tends to appear all convex or all concave. Thus, the globes (otherwise irrelevant to the experiment) help to see the stimulus as convex. Since earth globes are such familiar objects, they are not expected to distract attention. Finally, the short presentation time is insufficient to do the convex–concave flip, this tends to take conscious effort (and thus time) even for experienced observers.

The shading and the otherwise irrelevant earth globes also serve to evoke the awareness of sphericity in participants with only weak pictorial stereopsis. Of course, we cannot be certain that all participants actually experienced solid spheres. Some may have been aware of flat discs filled with texture in non-uniform motion. Maybe that is how they experience pictures of the globe. We are not aware of any (objective) method to avoid this possibility.

We obviously experimented with a variety of presentations before deciding on the present one. In retrospect, we are quite happy with it. There were no signs that observers met with ambiguities, they experienced the visual awareness of a ‘rolling ball’, painted on its outside surface. The question that interests us is whether they are able to ‘look inside it’.

Because we wanted to minimize the influence of reflective thought, each participant was presented with only one stimulus. Half of the observers were presented with pictures of the inward looking, the others with pictures of the outward looking scene. Although this is very inefficient in terms of observer’s usage, it avoids various possible artefacts.

In retrospect, this was probably unnecessary. When we explained the physical configuration, the photographic process, the display of the picture to people, it did not change the observer’s visual awareness. This equally applies to the authors themselves.

#### Responding

**Figure 6.**
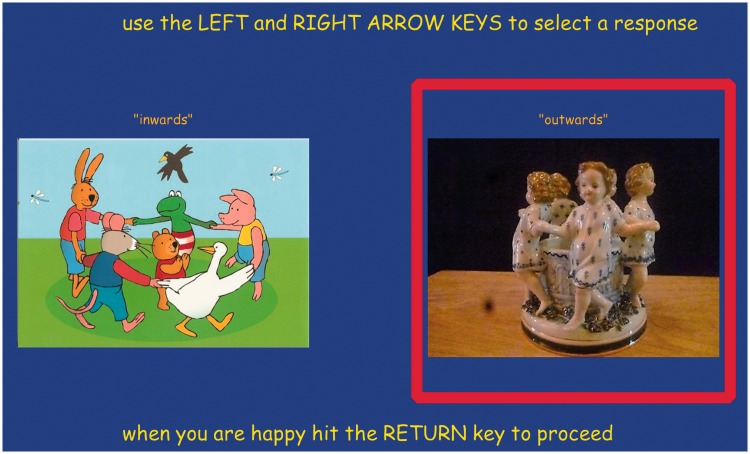
A screen shot in responding mode. The right picture has been highlighted.

The responding phase is a critical one because it is only too easy to introduce some systematic error. We used a full screen presentation for the response phase (Figure 6). It contained some text and the same two images as used in the instruction phase, again labelled as [inwards] and [outwards]. As the screen came up, these images are in random left/right order (independent of the other used in the instruction phase) and neither of them highlighted.

The screen had a top line [use the LEFT and RIGHT ARROW KEYS to select a response] and a bottom line [when you are happy hit the RETURN key to proceed]. Hitting the left or right arrow keys highlighted the corresponding left or right image. The participant was free to toggle arbitrary times, the response was concluded with a hit of the return (or enter) key.

*The rationale* was as follows. As the screen comes up, the participant becomes aware of the choices but is in no way led to some particular one. As the presentation is still mentally present, the response can be given on the basis of pictures alone. No doubt some participants translated everything in verbal terms, but this was by no means necessary. We tried to promote promptness to avoid the latter but to limit the response time appeared counterproductive. In practice, all participants reacted promptly, as we hoped they would. If some observers would have taken a much longer than median response period, we would have excluded them from the analysis, but this proves not necessary.

The random left/right order of the images was again recorded on the response file, for later checks, although it was not anticipated to be relevant.

#### Questioning

**Figure 7.**
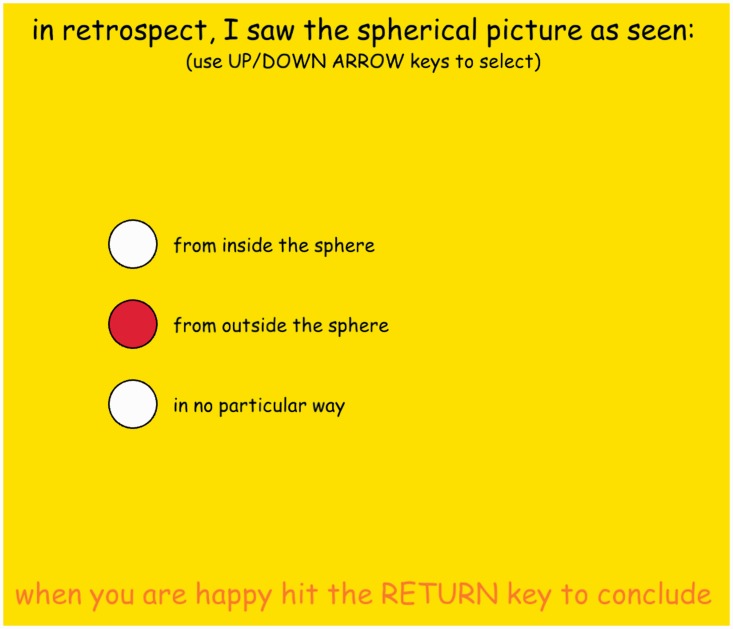
A screen shot of the questioning mode. ‘from outside the sphere’ has been selected.

In the questioning phase, we devoted the full screen to only a single question (Figure 7). It was formulated on top of the screen: [in retrospect, I saw the spherical picture as seen:] and (in smaller type) [(use UP/DOWN ARROW keys to select)].

Below this text, the screen had three circular discs with text labels (from high to low) [from inside the sphere], [from outside the sphere] and [in no particular way]. None of these was selected as the screen came up. Using the up/down arrow keys allowed scrolling through the categories. There was no time limit, but we promoted promptness. In practice, responses were indeed quick in all cases.

At the bottom of the screen was the text [when you are happy hit the RETURN key to conclude]. Hitting the return (or enter) key exited the program, after writing all relevant data to the response file.

*The rationale* was as follows. We did not want to inject any suggestion, so there was no initial highlighting. Most participants did not anticipate the question but did not take the time to think it over.

Of course, the meaning of the responses is necessarily ambiguous. However, it was decided that more detailed questioning would probably not serve to illuminate the nature of immediate visual awareness, but rather reflective thought in retrospect.

#### Post-experiment briefing

Although we did not formally plan a post-experiment briefing, indeed this happened in all cases. Participants had more to say than what they could respond in the experiment.

We were especially interested in whether they had been aware of a solid sphere, a concave hemispherical cup, a flat disc filled with moving texture or perhaps something else altogether. However, we avoided leading questions and merely took note of what was freely offered to us.

### Data

Each session took only a few minutes and contributed a line to the data file containing name, epoch, instruction order, scene type, response order, response of the participant and answer to the question in csv format.

**Figure 8.**
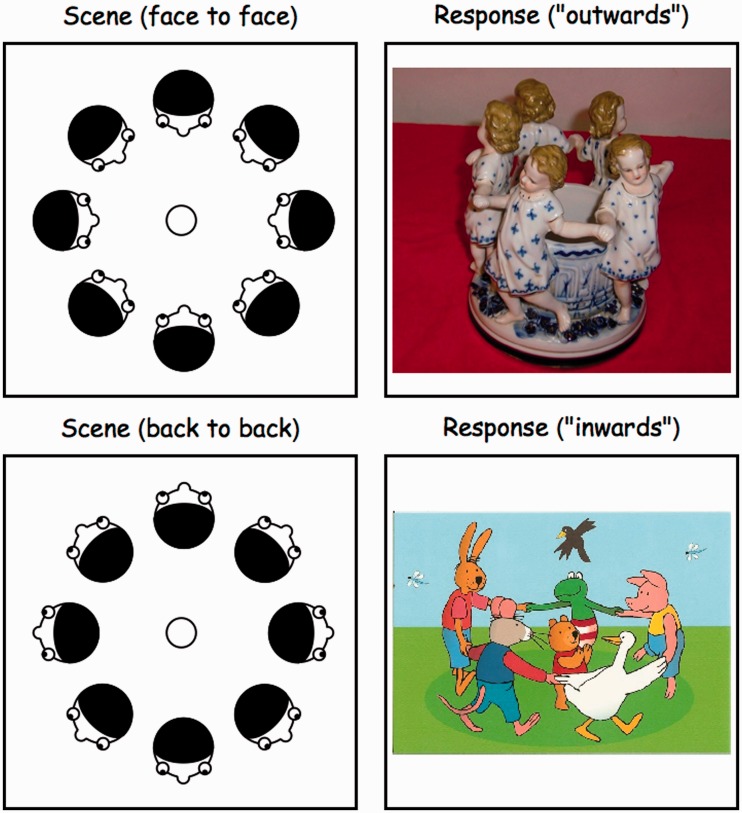
‘Looking inwards’ to become ‘face to face’, ‘looking outwards’ to become ‘back to back’ for the case of the scene. This explains the terms ‘inner’ and ‘outer’ for the scenes that were photographed (the left column) and for the responses (the right column). The two rows depict the most common cases encountered in the experiment, showing that the responses are ‘not veridical.’

The most important data are scene type and response. Both scene types and responses are either inwards or outwards, although the meaning of these words is obviously different in the two cases (Figure 8). Thus, in the coarsest approximation, the results can be summarized by a 2 × 2−matrix (one row per scene and one column per response):

**Table:** 

	Inwards	Outwards
outwards	36	5
inwards	7	37

In view of the observation that this matrix is nearly diagonal, one might be inclined to summarize even further to ‘73 different, 12 same’.

In response to the question we find:

**Table:** 

	Inside	Outside	Don’t know
non-veridical	4	63	6
veridical	7	5	0

Of course, there remains much more to check and correlate, but – in a sense – this sums up the major observations.

## Analysis

**Figure 9.**
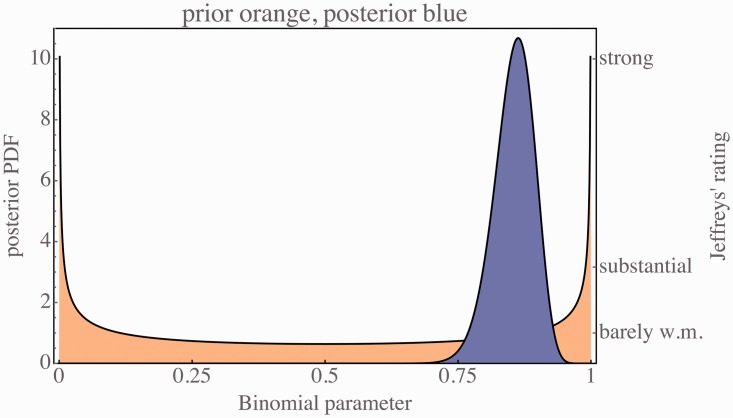
The posterior distribution (in blue) and Jeffreys’ prior (in orange). The influence of the choice of prior on the posterior distribution is very small.

So what does a finding of ‘73 different, 12 same’ imply? We use the conventional Bayesian analysis for the case of the unfair coin tossing problem.^1^ The prior and the posterior distributions are shown in Figure 9.

The maximum a posteriori estimate for the binomial parameter *p* is 0.86.^1^ The corresponding Bayes factor is 3.5 bits. On the scale suggested by Jeffreys,^2^ this is ‘strong’ evidence in favour of the binomial distribution with parameter 0.86 as compared with the fully non-informative prior. A 5% credible interval for the parameter *p* is {0.78,0.93}.

The Bayes factor for the comparison *p* > .5 versus *p* < .5 is 3.5 bits (again, ‘strong’).

The distribution over the inwards and outwards looking presentations does not yield an incentive to look for a difference.

Note that 6 of 85 participants answered the question with a [don't know], somewhat unequally distributed over participants with a veridical or a non-veridical answer. Because of the small numbers, there is not much to analyse here. The don’t knows are expected, because very likely many observers respond without any introspection going on.

For those 12 who responded veridically, 7 answered the question with [inside]. Perhaps they experienced a hollow hemisphere, or perhaps they indeed placed their mental eye inside the sphere, there is no way to know.

For those who answered the question non-veridically, 63 answered [outside], 4 [inside], 6 [don’t know]. Ignoring the [don’t know]’s, we find that the maximum a posteriori estimate of the probability of responding (outside) is 0.95, with a Bayes factor of 3.4 bit, ‘strong’ on Jeffreys’ scale.

### Conclusions

The conclusions are rather clear-cut. Given the Bayes factors, the rational inference is that observers experience a rotating spherical picture as seen from its exterior and that they do not mentally place themselves in its interior.

If observers had no trouble seeing the scene as it was, the posteriori distribution would have been vastly different. Even a fifty–fifty distribution would have been surprising. Instead, we find that observers systematically tend to have the non-veridical awareness.

The reason is also evident: *Observers locate their mental eye outside the spherical picture*. This is hardly surprising, since the mental eye is typically outside of any pictorial content. If that was not the case, the eye would be able to see itself! But nothing can be simultaneously in physical and in pictorial space, the objects in these spaces are ontologically distinct. For instance, although you can see your eye in a mirror, you still cannot see your eye, you only see a Doppelgänger. The mirror world is indeed *optically specified*, whereas the interior of the rolling ball is not.

## Discussion

The fact that our viewers tended to view the sphere from an exterior perspective may reflect our general visual diet in which pictures of all kinds are normally read from ‘the outside in’. Pictures are evidently material objects made of paper, ink or pixels, and we will naturally perceive them as such. But we also perceive their depictive content, with no apparent conceptual confusion. Pictures, therefore, have a remarkable dichotomous nature, being perceived as two quite separate things at the same time (Koenderink, van Doorn, Pinna, & Pepperell, 2016; Pepperell, 2015).

But pictorial conventions that have been in force since at least the Medieval period, in the European tradition at least, have implicitly located the beholder outside the picture space – as an external observer (Arnheim, 1982). Panoramas and ‘peepshows’ such as the view of Delft by Carel Fabritius in the National Gallery, London (1652) are rare cases of artists who deliberately attempted to locate the beholder’s experience inside the pictorial space. While such devices do not negate the pictorial dichotomy (we are rarely fully deceived by trompe l’oeil effects, even in virtual reality) they can profoundly alter the ’pictorial contract’ in favour of the content at the expense of the material.

**Figure 10.**
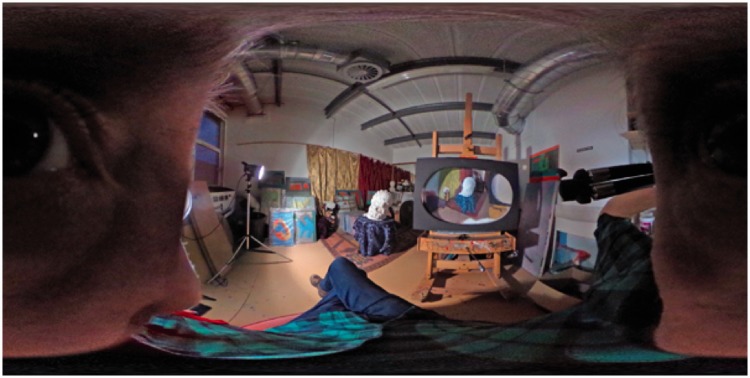
A 360° photograph of the artist’s studio (equi-rectangular map). When the photograph is viewed conventionally, the eye on the left seems to be directed at us, whereas in fact it is directed at the plaster bust of Hercules in the centre of the image. This illustrates how viewing the picture from an external perspective can be topologically misleading. Viewed from very close quarters, however, the photograph takes on a subjective aspect in which we may ‘inhabit’ the picture space, as though seeing the studio from the photographer’s point of view. This example illustrates the difference between looking ‘at’ and looking ‘as’ a picture. (Image by Robert Pepperell and Alistair Burleigh; a high-resolution version available at the publisher’s site.)

This difference can be illustrated with a 360° photograph made by one of the authors and a colleague Alistair Burleigh (Figure 10). Viewed conventionally, that is, from an external perspective, the content is readable but topologically misleading. We are inclined to mistakenly interpret the eye on the left as directed towards us, when it is actually directed at the plaster bust of Hercules in the centre. If, however, the image is sufficiently enlarged (a high-resolution digital file is available on the journal website) and viewed with one eye in line with the bust at a sufficiently close range, we are, with some effort, able to experience a strong sensation of being inside the pictorial space. It may even be that, given the presence of visual cues such as the photographers’ legs and nose in the picture periphery, we will experience something of the subjective viewpoint of the photographer itself. In sum, we are looking as ‘from inside’ the picture rather than ‘at it’ (Pepperell, 2017).

Many representational pictures can be viewed in this way; try looking very close up with one eye at a painting next time you are in an art museum, it is often a revelation. But the inability of our eyes to focus on very close surfaces and the nervousness of museum guards are usually discouragements.

Thus, it is perhaps not surprising that the majority of responses are not veridical. Most observers were aware of viewing the rolling ball from the outside as a pictorial ‘solid’ object. There is a priori no cue that such an object might depict a viewing sphere, after all the viewing sphere is never ‘seen’ as such in daily vision – it is not something that can be seen, being essentially *beyond* visible or invisible. In short, the ‘rolling ball’ presentations fail when seen at a glance, they are flawed as visual designs.

Conceptually, one deals with a peculiar type of topo-agnosia. It adds to the topo-agnisias detected in our earlier work on the planospheric depictions (Koenderink & van Doorn, 2017) in which observers failed to take the periodic nature of the horizon into account and failed to recognize instances of antipodal positions of pictorial objects. Thus, one has to reckon with (at least) three distinct forms of topo-agnosia that affect the intuitive, immediate awareness of full visual field depictions.

The particular topo-agnosia detected here is related to a number of phenomena that have been noticed and discussed for many years, even centuries.

Early instances relate to the topology of the surface of the earth and that of the sphere of heavens. This is tied up with the idea of ‘globes’ as representations (Stevenson, 1921).

Our immediate awareness of the surface of the earth is as that of an articulated plane, an impression that is especially strong on the open seas, in Siberian tundras, Utah salt flats and so forth. Pythagoras in the 6thc. bce and Parmenides in the 5thc. bce stated that the Earth is spherical. In the 2ndc. bce, Crates of Mallus devised a terrestrial sphere. Erathostenes (ca. 276bce–195/194bce) famously measured the radius of the earth, thus must have possessed an explicit idea of its spherical shape. This was the time terrestrial globes were available.

Indeed, you need to have a globe in mind before you can set out to find its radius. From a psychological perspective, the availability of globes must have been instrumental in convincing people of the spherical shape, because it allowed them to get off the surface and view the earth from a distance. The globe is as effective as a space vehicle, although the ‘The Blue Marble Shot’ of the earth (nasa archive AS17–148–22727, 7 December 1972 taken by the Apollo 17 crew) did not fail to create a sensation only decades ago. Were people finally convinced?

Well, some not, for even today, the insight that one inhabits the surface of a sphere is not universal, because the intuitive impression of a planar environment is so dominating. There is still belief in the ‘flat earth’ and there is even a widely shared belief in the ‘hollow earth’ (or sky-centrism). Reputedly Adolf Hitler and the Germain Kriegsmarine were believers (Kuiper, 1946; Lang, 1938) and sponsored expeditions to spy on the British navy by pointing infrared cameras to the sky near Peenemünde.

**Figure 11.**
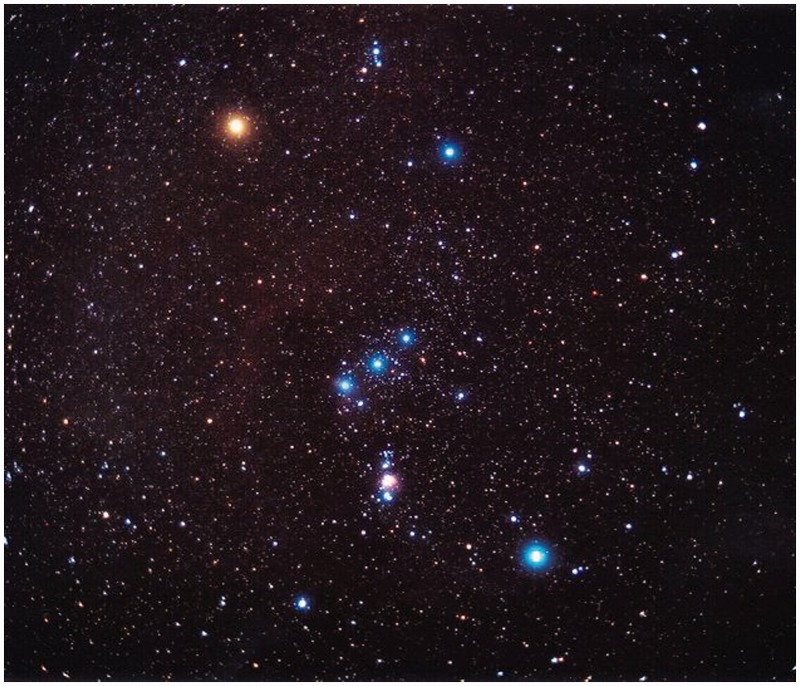
Photograph of the constellation Orion in the night sky, thus evidently a view from the inside. Betelgeuse is the bright yellow star at top left, Rigel is the bright blue star at bottom right. Together with the blue star Bellatrix at upper right and the white Saiph at lower left they subtend an easily recognized quadrangle. The three blue stars ‘in the belt of Orion’ at centre (Alnitak, Alnilam, and Mintaka) make that the constellation can hardly be confused with anything else. The slope of the belt in the quadrangle is a convenient sign of the orientation: If it slopes along the Saiph–Bellatrix diagonal you are ‘looking from the inside.’

More interesting is the case of the celestial globe, because the heavens are invariably seen from the inside (Figure 11), whereas celestial globes are invariably seen from the outside or their outside surfaces. Thus, there immediately arises a question of orientation or handedness.

**Figure 12.**
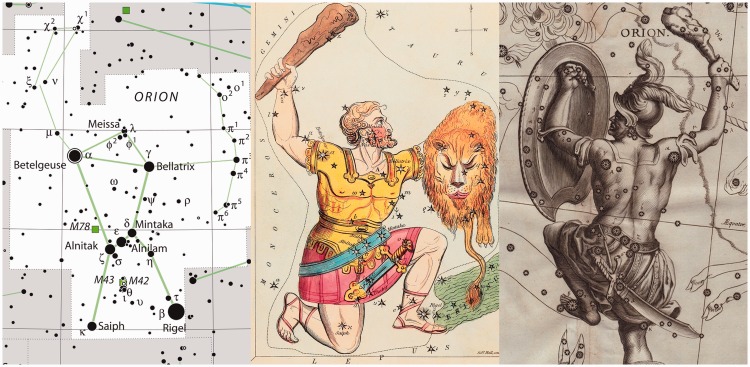
A composite of three maps of the stellar constellation Orion. At left is a modern celestial map, it is drawn as seen from the inside. Note that the basic Gestalt of Orion is made up from the four bright stars: Betelgeuse, Bellatrix, Rigel, and Saiph in an almost parallelogram, with a striking configuration of three collinear stars (the ‘girdle’, Alnitak, Alnilam and Mintaka) near the centre. At centre and right, two star maps of different orientation. The maps are mirror reversed and the figure of Orion is either seen ventrally or dorsally.

Perhaps not surprisingly, there exist two types of celestial globes and star maps, one a mirror image of the other (see Figure 12). This goes back a long time. The famous atlas *Kitab suwar al-kawakib* (published around 964ce, several copies extant) by Abd al-Rahman ibn ’Umar al-Sufi (903ce–986ce) shows the constellations in *both* configurations (Goldstein & Hon, 2007). On antique globes, where the personifications of the constellations are depicted, the figures are sometimes seen in dorsal (‘seen from the outside’) and sometimes in ventral view (‘seen from the inside’). From the lettering, it appears that some globes were routinely viewed in a mirror. Sometimes the celestial sphere is drawn on glass, so inside and outside are seen simultaneously. Clearly, users of celestial globes were aware of the topological problem and must have tried to transfer their mental viewpoint from outside to inside and perhaps vice versa. It remains an unsolved (unsolvable?) problem of visual design.

The notion that the correct viewpoint was *inside* the solid pictorial sphere apparently did not intuitively occur to our observers. This indicated that the popular ‘rolling ball’ paradigm is not very well suited to create a useful impression of a wide scene wrapped around the spectator’s eye. The popularity of this representation is possibly due to the interactive ‘rolling’ feature, but this is a mere gimmick that does in no way solve the problem.

**Figure 13.**
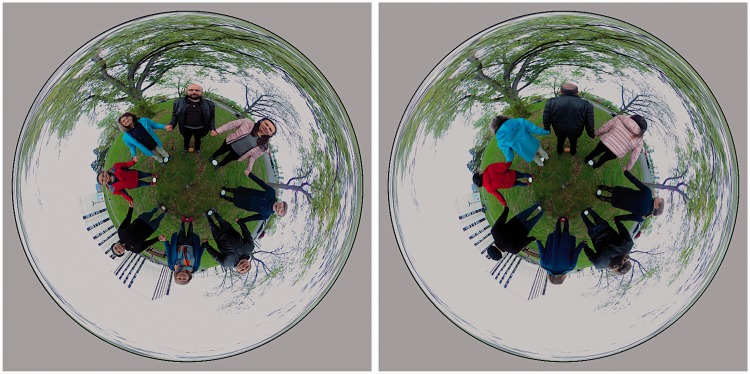
A so-called small earth representation is really a Riemann normal coordinates map centred on the nadir. The nadir is at centre of the picture and the radius is proportional to the angular distance from the zenith, that is $90^{\circ }+\vartheta$. The angle (clock-face direction) is the azimuth ϕ. The zenith is at 180° from the nadir, thus maps on the full circumference of this circular picture, a very singular feature of this map. The map is only conformal at the centre, although distortions are not too objectionable over much of the disc.

**Figure 14.**
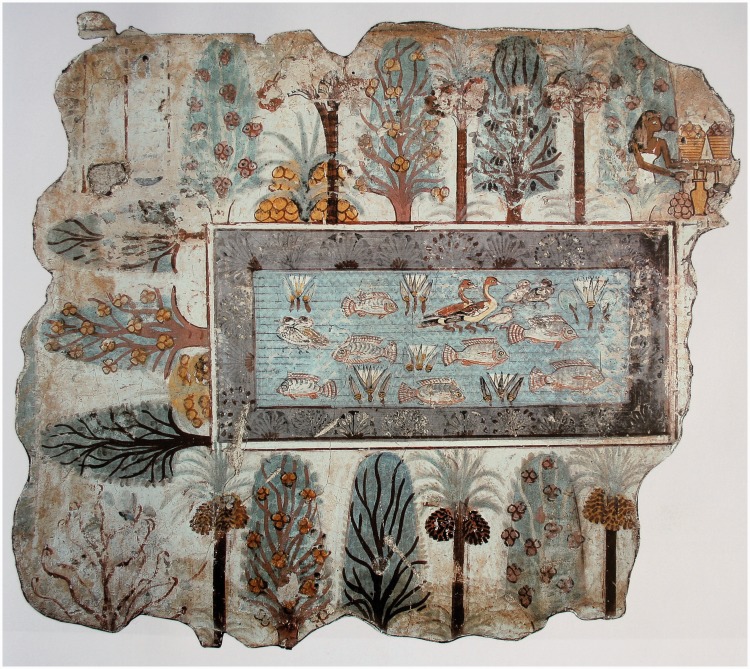
The Garden, fresco from Nebamun tomb, originally in Thebes, Egypt (circa 1380bce), now in the British Museum, London, UK. Painting on plaster, 72 × 62 cm. ([copyright] Yann Forget/Wikimedia Commons).

A much better impression is obtained – even in static representations – through more appropriate types of depiction. For instance, the ‘small earth’ maps shown in Figure 13 leave no doubt as to the topology of the scene, it is evident at a glance. Similar representations have been used even before our common era (Figure 14). These are very effective, despite containing no conventional linear perspectival depth cues. Instead, individual objects are represented from a ‘canonical’ perspective. In this format, the viewer is neither inside the scene nor outside it since there is no single implied viewing position. Despite this, the space being presented is quite legible.

Full spherical representations are rare in the visual arts. Partial representations are common enough, think of panoramas or frescoed domes. These are always seen from the inside, so it is easy to represent the optic array. Small paintings for display have to be painted on globes seen from the outside though. Few painters have made a career out of that, an exception is the contemporary artist Dick Termes (1991). He writes

When I am painting, however, it is easier to think of myself inside a transparent sphere, painting the world I see outside … I later imagine taking the sphere off my head and seeing from the outside what I mentally painted from the inside. Some of the larger spheres that end up in an outside environment are actually painted on the inside and viewed from the outside. (p. 290, 291)

So the ‘Termespheres’ are like our photographs. This may yield the type of uneasy inversions that troubled the observers in the experiment (again, Termes, 1991):

Another of my spheres seems at first to provide a look at a fishbowl, but, later, observers realize that they are inside the bowl, seeing the fish outside as well as the table and room in which the bowl is sitting. (p. 292)

In conclusion, our experiment shows that modern photographic and computer graphics technology have brought us yet another instance of a perceptual quandary that has plagued humanity since the dark ages. The usual brochures or websites used by the public to select a holiday destination or hotel will have to depend on a ‘quilt’ of mutually independent perspective renderings (photographs) for the foreseeable future – although interactive rolling ball and pannable planispheric depictions are already frequently encountered.

We conclude with a statement requested by a reviewer of this article: *This experiment tells us absolutely nothing novel about the visual system*.

Instead, it is about the intuitive impressions in viewing pictorial representations of physical scenes.

## Supplemental Material

sj-vid-1-ipe-10.1177 2041669518774806 -Supplemental material for View From Outside the Viewing Sphere Click here for additional data file. Supplemental material, sj-vid-1-ipe-10.1177 2041669518774806 for View From Outside the Viewing Sphere by Jan Koenderink, Andrea van Doorn and Robert Pepperell in i-Perception

## Supplemental Material

sj-vid-2-ipe-10.1177 2041669518774806 -Supplemental material for View From Outside the Viewing Sphere Click here for additional data file. Supplemental material, sj-vid-2-ipe-10.1177 2041669518774806 for View From Outside the Viewing Sphere by Jan Koenderink, Andrea van Doorn and Robert Pepperell in i-Perception

## Notes

1 We use the standard Bayesian analysis of the (unfair) coin tossing problem. A suitable prior is that suggested by Jeffreys, the beta distribution with shape parameters {1/2,1/2}, or, explicitly,

(1) $$J(p)=\frac{1}{\pi \sqrt{p(1-p)}}$$

where 0 < *p* < 1. The precise form of the prior is hardly of influence on the results, a uniform prior would do as well, for instance. It would make no difference to our conclusions. Using the binomial distribution, the posterior is

(2) $$P(n,m,p)=\frac{p^{m}\Gamma (n+1)(1-p{)}^{n-m}}{\sqrt{(1-p)p}\Gamma (m+\frac{1}{2})\Gamma (-m+n+\frac{1}{2})}$$

for *n* = 73, *m* = 12. The maximum a posteriori estimate for the binomial parameter *p* is

(3) $$p=\frac{2m-1}{2n-1}$$

(In our case 0.86.)

2 The scale suggested by Jeffreys (1939) is:

**Table:**

Bayes factor	Strength of evidence
<10°	Negative
10^0^ to $10^{1/2}$	Barely worth mentioning
$10^{1/2}$ to 10^1^	Substantial
10^1^ to $10^{3/2}$	Strong
$10^{3/2}$ to 10^2^	Very strong
>10^2^	Decisive
